# The Foreign Journals: Pathology, Practical Medicine, and Therapeutics

**Published:** 1839-07

**Authors:** 


### MEDICINE.

### *On the Communication of Phthisis Pulmonalis to Domestic Animals.* By Dr. Malin, of Lübbenau.

[We give the following narrative as we find it: without doubting the statement, we refuse to admit the inference.]

A man, fifty-eight years old, labouring for several years under a pulmonary consumption, had a house-dog which licked up, with great eagerness, his purulent sputa. In half a year the dog became affected with a cough and expectoration of matter, and at last died completely emaciated. Another, a wolf-dog, one year old and one foot high, was procured. This likewise commenced to lick the nauseous mess, although it got milk and flesh. In the course of half a year it also sickened and died within twenty weeks.

To convince himself, Dr. Malin opened the thorax. Both lungs were almost completely destroyed by suppuration. In the right there was a large closed vomica. *Wochenschrift für ges. Heilkunde. April* 6, 1839.

---

### *Remarks on the uncertainty of the diagnostic signs of Albuminuria.* By A. Toulmouche, M.D. &c.

[M. Toulmouche relates two cases, observed by himself at the infirmary of the prison at Rennes, for the purpose of showing, on the one hand, that the urine may be strongly albuminous, and other alleged symptoms of Bright's disease be present during life, and yet no morbid change be discovered in the kidneys after death; and, on the other, that the renal lesion may exist without producing any of the symptoms presumed to be its necessary consequences. The case adduced in support of the latter position, is extremely defective in its details, and proves nothing but that M. T. occasionally contents himself with a very slovenly examination of his patients. The other is decently reported, though the state of the kidneys should have been more fully described, and adds to the list of facts of similar character already accumulated: we subjoin the more essential particulars of this case.]

Dano, æt. forty-three, admitted June 26, 1837, with diarrhœa and gastralgia; the former of which continued, in spite of treatment, during a part of the month of July. *July* 12.—The patient began to cough, and œdema of the inferior extremities to appear; she had no fever, but complained of pain in the loins and limbs, which were presumed to be rheumatismal, and treated by the vapour-bath. During the month of August, the œdema sometimes increased and sometimes appeared to diminish with the lumbar pain. *Sept.* 9.—Pain in the side, treated as pleurodynic: on the 20th, the heart was ausculted, but no abnormal condition discovered. *Oct.* 20.—The habitual pain in the loins, and progressively increasing œdema, as well as the deposition of a thick stratum of albumen from the urine, when treated by heat and nitric acid, (on the 23d it is noted as being three or four lines thick,) led to the diagnosis of Bright's disease. The medicines hitherto employed had been diuretics, tonics, anodynes, &c.; dry cupping in the loins and the use of the vapour-bath were now prescribed. The patient was unable to bear the latter more than three times; and on the 18th *Nov.* the œdema having meanwhile reached the abdomen and fore-arms, she expired in a state of tranquil coma. *Inspection twenty-two hours after death. Brain.*—An effusion of blood about the size of a large hemp-seed in the centre, nearly of the right hemisphere, the cerebral substance around it tinged yellow, some limpid serosity between the arachnoid and pia mater. *Thorax.*—Two pints of bloody serum in the left pleura, pseudo-membranes; cicatrices of caverns in the summits of both lungs, which are œdematous, miliary tubercles, bronchial membrane red; heart small, its tissue pale, polypiform concretion filling right ventricle. *Abdomen.*—Two pints and a half of mily serosity in peritoneum, firm, dense, membranous adhesion of a portion of the ilium to the bladder; ulcerations through the entire tract of the small intestine, most numerous in the neighbourhood of the cæcum, the fundus of some of these ulcerations is studded with miliary tubercles; vast ulceration in the ascending colon; spleen small, shrivelled, and firm; liver of ordinary size and healthy, numerous calculi in the gall-bladder; kidneys of very small size and perfectly healthy, both substances pale; bladder contains a good deal of limpid urine, its coats thin and healthy. *Gazette Médicale. Fevrier,* 1839.

---

*On Chlorosis accompanied by Menorrhagia.* By M. Trousseau.

The coincidence of diminished or suppressed menses with chlorosis is so common, that the fact is frequently overlooked that a chlorotic state may exist in conjunction with menorrhagia; in both we have extreme paleness, discoloration of the blood, a tendency to dilatation of the heart, *bruit de soufflet* in the principal arteries, and neuralgia in different regions; when the menstrual flow is deficient, these symptoms are said to arise from chlorosis, when it is superabundant, from anemia. We frequently find that chlorosis may be traced in the first instance to a copious nose-bleeding or to any other unusual loss of blood, arising accidentally or produced therapeutically; so that a transitory anemia will give rise to permanent discoloration and increased liquidity of the blood. This altered state of the circulating fluid is itself sufficient to give rise to hemorrhage, which, thus occurring alternately as cause and effect, does not allow the patient to escape from its evil consequences. This form of chlorosis is however much less frequent than that which is attended by amenorrhœa; it forms only one fourth of the cases occurring in adults, and perhaps one twelfth in those of young girls. In twelve cases collected by M. Trousseau, there was no serious lesion of the uterus. It would appear at first view that the treatment adopted for amenorrhœal chlorosis would not be suitable for the menorrhagic form; thus, iron, which is given with so much success to restore the menstrual secretion, would hardly appear appropriate when that secretion is in excess: but is not the effect of iron on chlorotic patients rather tonic than emmenagogue? We generally find the health partially reestablished before the menses return: the complexion regains its natural tint, the depraved appetite, the pain at stomach, the palpitation of the heart cease, and the *bruit de soufflet* in the arteries is lost, so that the patient frequently recovers the appearance of health before the menses reappear. Presently this secretion is reestablished, in consequence of the general return of the system to a healthy state. Iron then is only emmenagogue because it is tonic or reconstituent. Reestablished health is not owing to the returning menses, but the contrary. On this view we shall have no difficulty in conceiving that preparations of iron will be of the greatest utility in menorrhagic chlorosis, in which it will operate both as tonic and hemostatic. Giving freely, the preparations of iron between two menstrual periods, we shall find the blood rapidly regain its lost constituents of colouring matter and fibrine, and the secretion will be much less abundant but more coloured. If more powerful anti-hemorrhagic means are required, the ergot of rye will be very useful. Uterine hemorrhages are generally more violent in the night than during the day, and they occur with the greatest violence about four or five o'clock A.M. Without pretending to account for this singularity, we shall find it highly useful to give a dose of *recently-powdered* ergot (gr. xv. to ℈j.) in the evening, and again about four in the morning. In many cases, the more simple and agreeable administration of acids will be sufficient; of these, the best is the citric, given in its natural form of lemon-juice. If these means are necessary, the chalybeate must be suspended during the menstruation, and afterwards continued whilst any symptoms of chlorosis remain.

*Journal des Connaissances Medico-Chirurgicales. Dec.* 1838.

*On Ligature of the Limbs, as a means of shortening the duration of the paroxysm of Intermittent Fever.* By DRS. PENBECK and GOEDECHEN.

[THE following cases contain no novelty; but it is well occasionally to recall the attention to important facts in pathological and practical medicine.]

CASE I. A man, aged fifty, of robust constitution, had been suffering for more than three months from tertian fever, which had resisted all rational means of treatment. Dr. Penbeck, therefore, advised the patient to try the application of the ligature. As the sensation of cold had hitherto commenced in the feet, and had from thence spread over the rest of the body, the ligature, as soon as the first symptom of the cold fit was perceived, was applied tightly immediately above the knee; the cold was, in consequence, soon dissipated, but some heat followed with rather profuse perspiration. The patient continued to apply the ligature immediately on the slightest feeling of cold, at the usual period for the return of the paroxysm, and succeeded in arresting the further development of the cold fit. Heat and perspiration, however, still continued; but these symptoms were removed by quinine, which had formerly been given without relief.

CASE II. A woman, aged fifty-two, was attacked with intermittent fever, and refused to take any medicine for its removal. Recourse was, therefore, had to the ligature as the only means which promised to be beneficial. The first application had scarcely any effect; the second shortened the cold fit and diminished the heat and perspiration. By each succeeding application the paroxysm became less severe, and disappeared entirely with the sixth, and in fourteen days the patient was in a condition to undertake a fatiguing journey.

CASE III. A sailor, aged thirty-seven, of weak constitution, was attacked in February, 1834, with septan fever, complicated with gastric affection. A treatment, principally directed against the latter, removed the fever, and the patient was dismissed the hospital. He was again received, on the 4th of April, with tertian fever; the cold fit lasted about one hour and a half, and was extremely severe; the hot and sweating stages, which were moderate in intensity, lasted respectively half an hour and a quarter of an hour. After some preliminary treatment, to remove some gastric symptoms, the ligature was applied, at the approach of the paroxysm, to the extremities, with the effect of reducing the cold stage to one hour's duration, and of changing the fever to its original septan type. Each succeeding application of the ligature reduced the intensity of the paroxysm, and in three weeks the patient was dismissed cured.

*Zeitschrift für die gesammte Medicin.* Band 9, Heft 1.

---

*On the Mechanical Action of Tartarized Antimony.* By G. POLLI.

ACCORDING to Giacomini the effects produced by the application of tartar-emetic to the skin do not in the least depend on its dynamic action, but on the mechanical action of its minute crystals. Hence the effect of emetic frictions on the economy must be quite different from that produced by the same salt taken internally. In proof of his opinion, Giacomini adduces the sharp angular form of the crystals and alleges that their action is increased by the addition of powdered sugar, that the same result will be produced by any other crystallized salt or even by glass, and that the aqueous solution applied in local baths causes no irritation. But, on the other hand, considering that the cutaneous efflorescence, produced by tartar emetic, invariably presents the same characters, that a similar eruption is frequently developed by sympathy in parts far distant from the seat of friction, such as the scrotum, neighbourhood of the anus, &c., and that pustulation follows when the ointment is simply spread on the skin, M. Polli inclined to the old opinion of its acting dynamically. To settle the question, he and several of his friends made a number of experiments of which the principal results were as follows: 1st. Tartar-emetic friction always produced a papular eruption, with tendency to pass into the pustular form, and in three, a similar affection or pruritus was observed at the genitals or anus. 2d. The eruption never appeared before the thirty-sixth or after the forty-eighth hour after friction. 3d. Simple friction, with cloths dipped in water, when performed where the sebaceous glands are prominent, produced, in about half an hour, an eruption of rosy papulæ, without any tendency to become pustular or to suppurate; where the skin was smooth erythema only followed. 4th. The repeated application of local baths, made with a saturated aqueous solution of tartar-emetic, never produced any eruption. 5th. Ointments made with the same proportion of sulphate of potass, or glass, sometimes produced a slight papular eruption about two days after the experiment, but in the majority of cases had no such effect, and in no instance where the skin was smooth. The eruption was always proportional to the violence of friction.

*Giornale delle Scienze Med.-chir.* No. xxv.

---

### *Cure of a Stubborn Case of Aphonia by means of Ammoniacal Vapours.* By Dr. Gerner.

A young lady was affected, in consequence of a cold, with complete loss of voice, which had already existed three months, notwithstanding all the remedies which were tried. Dr. Gerner, supposing the cause of the affection to be a relaxed state of the mucous membrane of the trachea, at last cured the patient completely in three days by the inhalation of ammoniacal vapours, disengaged from a mixture of a solution of muriate of ammonia and carbonate of potass.

*Zeitschrift für die gesammte Medicin, &c. Feb.* 1839.

---

### *On the Use of Dr. Blaud's Pills in Chlorotic Affections.* By M. Adome.

A medicine very much resembling the pil. ferri comp. of the London Pharmacopœia has acquired great celebrity in the south of France on account of the cures it has effected in cases of chlorosis. It bears the name of its inventor, Dr. Blaud, who is Senior Physician to the Hospital of Beaucaire. The formula which he gives is as follows:

*Sulphate of Iron, half an ounce.*
*Subcarbonate of Potash, half an ounce.*

Mix with *mucilage*, and triturate the mass to a proper consistence, and divide into *forty-eight* pills.

The objections to this formula are that the pills are excessively large; that they have a very repulsive odour; and, particularly, that a chemical change quickly takes place in the mass, the carbonate of the protoxyde being, after a short time, converted into the sesquioxyde of iron. Dr. Blaud, however, maintains that, whatever chemical changes occur, his pill is of equal advantage medicinally, and corroborates this statement by a long list of cases, in which a cure was obtained, generally, in three or four weeks. M. Adome, in a memoir presented to the Academy of Medicine, proposes to remedy the defects above mentioned by incorporating with the pills a portion of *sugar* and of *pulv. altheæ*. As soon as the pills are made, he rolls them on a plate moistened with a mixture of mucilage and sugar, and then covers them with a fine powder, composed of sugar and gum-arabic, aromatized with some essential oil, to correct the disagreeable odour. This process is repeated a second time, and the pills are then, in a great measure, protected against the influence of the oxygen of the atmosphere.

*Revue Médicale et Bulletin de l'Académie. Dec.* 1838.

